# Correlates of support for international vaccine solidarity during the COVID-19 pandemic: Cross-sectional survey evidence from Germany

**DOI:** 10.1371/journal.pone.0287257

**Published:** 2023-06-23

**Authors:** Florian Stoeckel, Jack Thompson, Paula Szewach, Sabrina Stöckli, Matthew Barnfield, Joseph B. Phillips, Benjamin Lyons, Vittorio Mérola, Jason Reifler

**Affiliations:** 1 The Department of Social and Political Sciences, Philosophy and Anthropology, University of Exeter, Exeter, United Kingdom; 2 Department of Consumer Behavior, University of Bern, Bern, Switzerland; 3 Dept of Marketing, University of Zurich, Zurich, Switzerland; 4 Department of Government, University of Essex, Colchester, United Kingdom; 5 School of Psychology, University of Kent, Canterbury, United Kingdom; 6 Department of Communication, University of Utah, Salt Lake City, Utah, United States of America; 7 School of Government and International Affairs, Durham University, Durham, United Kingdom; University of Naples - Parthenope: Universita degli Studi di Napoli Parthenope, ITALY

## Abstract

During the COVID-19 pandemic, many residents of high-income countries (HICs) were eligible for COVID-19 vaccine boosters, while many residents of lower-income countries (LICs) had not yet received a first dose. HICs made some efforts to contribute to COVID-19 vaccination efforts in LICs, but these efforts were limited in scale. A new literature discusses the normative importance of an international redistribution of vaccines. Our analysis contributes an empirical perspective on the willingness of citizens in a HIC to contribute to such efforts (which we term international vaccine solidarity). We analyse the levels and predictors of international vaccine solidarity. We surveyed a representative sample of German adults (n = 2019) who participated in a two-wave YouGov online survey (w1: Sep 13–21, 2021 and w2: Oct 4–13, 2021). International vaccine solidarity is measured by asking respondents preferences for sharing vaccine supplies internationally versus using that supply as boosters for the domestic population. We examine a set of pre-registered hypotheses. Almost half of the respondents in our sample (48%) prioritize giving doses to citizens in less developed countries. A third of respondents (33%) prefer to use available doses as boosters domestically, and a fifth of respondents (19%) did not report a preference. In line with our hypotheses, respondents higher in cosmopolitanism and empathy, and those who support domestic redistribution exhibit more support for international dose-sharing. Older respondents (who might be more at risk) do not consistently show less support for vaccine solidarity. These results help us to get a better understanding of the way citizens’ form preferences about a mechanism that redistributes medical supplies internationally during a global crisis.

## Introduction

In an increasingly globalized world, pandemics have the potential to spread to far more people than in the past. High levels of vaccination across the globe will be key to containing a number of them [1, 2]. However, some countries are better equipped than others to vaccinate their population. During the COVID-19 pandemic, we saw a situation in which many residents of high income countries (HICs) were eligible for COVID-19 vaccine boosters, while many residents of lower income countries (LICs) had not yet received a first dose. HICs were able to secure contracts for a disproportionate amount of vaccine supply. A new normative literature emphasizes the importance of international vaccine solidarity [1, 3, 4]. The development of international vaccine transfer initiatives such as the COVID-19 Vaccine Global Access (or COVAX) represents an important step in trying to address these disparities in practice [e.g. 5]. A key component of HICs being able to contribute to schemes that redistribute vaccines internationally is marshaling public opinion in favor of providing it.

Our analysis contributes to the empirical literature which focuses on levels of public support for international vaccine solidarity and the factors that shape citizens’ preferences [6–11]. We add empirical evidence by investigating the correlates of directing vaccine supply abroad. This is important to understand where public support and opposition to such schemes is coming from. This has policy implications for situations that require a process in which medical supplies are redistributed internationally. Germany is an interesting country case because it is one of the largest donors of foreign aid [12]. It has a robust welfare system with moderate levels of redistribution as well as support for domestic redistribution [13, 14].

One of the central issues we shed light on is whether attitudes in HICs towards sending COVID- 19 vaccines to LICs have the same etiology as attitudes toward foreign aid. Citizens could treat sending vaccines as fundamentally different from foreign aid. If a pandemic is raging throughout both the donor and recipient countries, the decision to deliver life-saving vaccines may appear unusually zero-sum and therefore rather unlike foreign aid (funds) which are often a small share of a nation’s budget. Any vaccine that goes into the arm of a low-income country recipient is not going into the arm of a recipient in one’s own country. Yet, some citizens already seem to see foreign aid as a zero-sum game. For instance, countries lacking robust welfare systems contain people who both want increased domestic spending and decreased allocation to foreign aid [15]. This implies that wanting one means discounting the other. If people already see foreign aid as zero-sum, the correlates of support for sending vaccines abroad will be fairly similar. People are also more willing to give foreign aid when they see their country as in a better position to lose resources [16]. An unequal international vaccine distribution might be approached by voters as a special and particularly acute case of global inequality, making it likely for citizens’ views to be driven by similar factors. We assume that this is the case even if foreign aid might invoke monetary transfers more directly than the redistribution of vaccine doses.

Existing work on support for foreign aid finds a few empirical regularities. For instance, when the public sours on foreign aid, foreign governments tend to invest less in it [17]. This happens both as a result of governments taking cues from voters and changes in party control that come from elections [18, 19]. People with the capacity to trust, identify with, and otherwise empathize with potential aid recipients tend to support aid more [20–23]. This is especially the case if the recipient seems “deserving” of that aid, whether due to material need or stereotypes about the recipient’s agency [22, 24]. Additionally, as a consequence of low numeracy, people tend to overestimate the level of foreign aid their country gives [25] and underestimate how good of a position their countries are in to give aid [16]. Once they know how little their country spends on foreign aid relative to its resources, people tend to be more supportive of foreign aid [16, 25, 26].

In recent years, both surveys as well as conjoint experiments have attempted to describe and untangle factors underlying vaccine solidarity preferences. Surveys have generally found overall support to be high; a plurality of people in a wide range of HICs support various international vaccine solidarity schemes aimed at LICs, albeit there is important variation [6, 10, 11]. Indeed, some studies even found preference for vulnerable populations in LICs over the respondent’s co-national recipients [9]. On the other hand, though, some studies have shown Germans in particular to display a preference for sharing schemes that include only HICs [8] or co-national recipients [9]. Preferences also appear to differ along ideological lines and related orientations [7, 11], with leftwing orientations being associated with higher support than rightwing ones in the US and Germany (but see [8]). Lastly, it seems self-interest may play a role [9], especially in terms of older respondents exhibiting less support for redistribution [9, 11].

We tested a set of pre-registered hypotheses on the correlates of international vaccine solidarity using a large, nationally representative panel survey in Germany. The survey was fielded before an increase in infections in the fall of 2021, though at a time when the supply of booster shots was limited [27, 28]. Our results demonstrate that public attitudes towards international vaccine solidarity are consistent with broader views concerning (global) inequality. We found that support or opposition to sharing vaccine doses with LICs is associated with similar factors to those predicting redistribution and foreign aid preferences [e.g. 15, 23, 29]. That is, citizens seem to understand the question of unequal COVID-19 vaccine distribution as a specific, critical instance of the broader question of inequality in the distribution of economic resources rather than a unique phenomenon.

### Hypotheses

What shapes redistribution attitudes more broadly? Research suggests worldviews, personality traits, and self-interest each likely contribute [22, 30–33]). Delton et al. [30], for instance, identify a mixture of ideology, compassion, and self-interest as key determinants of public support for such policies. Similarly, we would also expect these broad set of factors to predict preferences related to an international redistribution of vaccines. Here, we specify which particular political orientations, worldviews, personality traits, and markers of self-interest we expect to play a role. We present a series of hypotheses regarding factors associated with international vaccine solidarity below (pre-registration link: https://osf.io/4umzv).

### Political orientations and worldviews

Research in political economy sees citizens’ views on domestic redistribution as an expression of their personal income situations. Those with no or low incomes (i.e., who stand to benefit from redistribution) prefer more redistribution than those with high incomes (who stand to lose out financially from redistribution) [34]. However, citizens’ preferences for redistribution can also or in part be an expression of their considerations about inequality [15, 35]. Since those who support domestic redistribution prefer a more equal distribution of resources, we expect them to be more supportive of redressing current global inequalities in vaccine distribution, even if this does not have any bearing on their personal income level (in our preregistration, we use the term *ideological positioning* in the context of this hypothesis.) We also test this logic with a more general measure for citizens’ political positions, namely their left-right self-placement (but we do not insert both items into the same model to avoid multicollinearity). We expect that how people position themselves on a broader left-right political spectrum would be associated with support for vaccine dose sharing: those further to the left will tend to be more supportive.

Similarly, cosmopolitanism strongly shapes attitudes towards redistribution at the international level. Cosmopolitans generally see themselves as citizens of the world [36, 37]. People tend to allocate resources to in-group members more generously than outgroup members [38]. This matters also in the context of COVID-19, e.g. in a situation of scarce vaccines, citizens prioritise natives over immigrants [39]. Cosmopolitans are more likely to include people outside their country as ingroup members. This is because they appreciate “other human beings irrespective of their national origin” [36, p. 1762]. Hence, cosmopolitans are more willing to redistribute resources to countries in need, including poorer EU member states [36, 40–42] and poorer countries in general [23, 43]. As such, we expect cosmopolitans to be more supportive of international dose sharing.

### Empathy and self-interest

We expect that the psychological trait of empathy is linked to support for international dose-sharing. Empathy is characterised by experience, understanding, and interest in the feelings or welfare of other people [22, 44]. Higher empathy predicts higher support for foreign aid [22]. Therefore, we expect higher empathy to predict higher support for international vaccine solidarity.

Conversely, though, we pre-registered a hypothesis on the role of age. We believe age may function as a proxy for self-interest regarding COVID-19 vaccinations. Sharing doses with other countries limits the number of doses available domestically. It stands to reason that, to the extent that citizens factor in their own self-interest in forming their attitudes, those who stand to benefit most from vaccination will be least likely to support sending those vaccines overseas. Restricting the domestic vaccine supply might be seen as particularly risky among populations vulnerable to COVID-19, particularly older citizens [45, 46]. Therefore, we expect older citizens to oppose international vaccine solidarity.

### Research questions

In addition to these main hypotheses, we also explore an additional set of pre-registered research questions. First, we examine an additional worldview that has particular relevance to vaccine attitudes—conspiratorial thinking. Conspiratorial thinking captures an individual’s propensity to assume conspiratorial intent behind various events and policies. Citizens high in conspiratorial thinking tend to be more vaccine-hesitant [47]. Since vaccine hesitancy captures perceptions about the effects of vaccines for people in general, one might not expect a link between conspiratorial thinking and attitudes towards international dose sharing. However, conspiratorial thinking is concomitant with higher skepticism towards international organisations [48], which may preclude support for international collaboration regardless of personal beliefs about vaccines. We thus consider the possibility of an association between conspiratorial thinking and international vaccine solidarity.

Lastly, we examine a potential interaction between cosmopolitanism and empathy [31, 33]. The discussions above point to the possibility that the role of empathy might be dependent on the role of cosmopolitanism, and vice versa. Even though empathy predicts higher support for foreign aid, vaccines, unlike foreign aid, can benefit both the in-group and the out-group. Even highly empathetic people can choose to withhold their empathy from out-groups in favor of the ingroup, driving polarisation in perceptions of opposing partisans [49]. One such ingroup can be the national community. Therefore, among those lower in cosmopolitanism, who are less apt to count those in other countries as in-group members, empathy may lead to lower support for international dose-sharing. Conversely, among cosmopolitans that count people in other countries as in-group members, empathy may not lead to this kind of parochialism. We explore this possibility.

## Materials and methods

We conducted a two-wave online survey in Germany (Wave 1: September 13–21, 2021, N = 2,801; Wave 2: October 4–13, 2021, N = 2,019). In the second wave, respondents were 50.22 years old on average (SD = 17.07), 51% female, and 26% university educated. Ethical approval for this study was obtained from a UK Russell Group university (approval ID 489681). Informed consent was recorded before participants began the survey. Respondents were shown information about the study on an introductory screen that ended with the following statement: I voluntarily agree to participate and to the use of my data for the purposes specified above. Respondents expressed written consent by selecting an “I agree to participate” button. The authors did not have access to information that could identify participants. Questions relating to vaccine solidarity were asked in wave two. The survey fieldwork was conducted by YouGov with a representative sample. All results reported below were based on a weighted sample, using weights provided by YouGov. The composition of the unweighted sample can be found in S1 File.

### Measures

Our outcome measure was a variable that measures attitudes towards vaccine solidarity. Given the absence of an established measure when we prepared our survey questionnaire, we developed the following new question: "Coping with the COVID-19 pandemic requires difficult decisions. By the end of September, about 64 percent of people eligible for vaccination had been vaccinated at least once. What do you think is the more important priority now for the use of Germany’s vaccine stocks: offering a third vaccine dose ("booster vaccination") to people in Germany or giving vaccine stocks for first and second vaccine doses to less developed countries?"

Our survey was fielded when the number of daily COVID-19 cases was decreasing. This was also at a point well before the Omicron variant was discovered. This is important to note, because the trade-off that respondents faced was one between improving protection against COVID-19 (for themselves or others) or some initial protection for individuals in LICs. This was obviously different from a situation in which cases were rising or when a new variant spread, which might have meant that two doses offered little protection and a third dose was more essential. Nevertheless, the supply of booster shots was still limited both within Germany and much more so globally [27, 28]. Booster shots were only available to larger groups of the population later in Germany. We believe however that these issues would affect baseline levels of public support for international vaccine solidarity, though, not the factors that shape citizens’ views (which is the focus of our analysis).

We measured ideological orientations in two ways. Our main measure (support for domestic redistribution) asked respondents whether the government should do more to reduce income inequality on a 5-point scale from 1 (strongly agree) to 5 (strongly disagree) [50, 51]. Our secondary measure required respondents to place themselves on an 11-point scale from 0 (very left) to 10 (very right).

To measure cosmopolitanism, we asked respondents whether they believe that globalization threatens Germany’s identity on a five-point scale from 1 (strongly agree) to 5 (strongly disagree) [52]. We also used immigrant sentiment and authoritarianism as alternative measures of cosmopolitanism in S1 File.

To measure empathy, we assessed respondents’ agreement with an item from a common empathic concern scale [53], with responses ranging from 1 (strongly agree) to 5 (strongly disagree): "When I see a person being taken advantage of, I want to protect them".

We divided respondents into four age groups: 18–24, 25–44, 45–54, and 55+. The youngest cohort was our reference category in all models, but we examined the robustness of findings to changes in how we coded age.

To measure conspiratorial thinking, we assessed respondents’ agreement with three items [54] and a 5-point scale from 1 (strongly agree) to 5 (strongly disagree). An example item is: "Much of our lives are being controlled by plots hatched in secret places".

All question wordings and coding decisions (also for the control variables gender, education, partisanship, and social class) can be found in S1 File.

## Results

### Descriptive results

Overall, we find that 48 percent of respondents prioritized giving available doses to citizens in LICs, 33 percent preferred these doses to be used as boosters domestically, and 19 percent of respondents selected the "don’t know" category (after employing weights).

We tested our hypotheses using logistic regression models. While our pre-registration specified OLS regressions, we relegated these to S1 File (see S3 Table in S1 File) in favor of logistic regressions with odds ratios. Table 1 shows results for four models. The leftmost column shows our main model (model 1). Each of the other models built on the main model save for making a single change. In the second model, left-right self-placement was used as a measure of ideological orientation instead of support for domestic redistribution. In the third model, there was an added cosmopolitanism x empathy interaction. The fourth model added controls for party affiliation (which other literature has linked to citizens’ views on international vaccine solidarity [7, 11]).

**Table 1:** Results of logistic regression models (coefficients show odds ratios).

	*Support for international vaccine solidarity*			
	Model 1	Model 2	Model 3	Model 4
Cosmopolitanism	1.279***	1.252***	1.175	1.237***
	(0.061)	(0.061)	(0.137)	(0.062)
Empathy	1.272***	1.281***	1.202	1.275***
	(0.069)	(0.072)	(0.109)	(0.072)
Age 25–44	0.826	1.105	0.825	0.778
	(0.160)	(0.223)	(0.160)	(0.154)
Age 45–54	0.813	1.171	0.811	0.859
	(0.171)	(0.256)	(0.170)	(0.184)
Age 55+	0.581*	0.788	0.580*	0.635
	(0.111)	(0.157)	(0.111)	(0.124)
Conspiratorial Thinking	1.096	1.163**	1.099	1.078
	(0.058)	(0.063)	(0.059)	(0.060)
Support for Domestic Redistribution	1.307***		1.303***	1.253***
	(0.067)		(0.067)	(0.069)
Left-Right Self-Placement		0.856***		
		(0.028)		
Female	1.073	1.022	1.074	1.057
	(0.104)	(0.102)	(0.104)	(0.107)
University Education	1.193	1.063	1.193	1.198
	(0.134)	(0.120)	(0.134)	(0.142)
Social Class	1.014	0.993	1.013	1.035
	(0.035)	(0.035)	(0.035)	(0.038)
Cosmopolitanism * Empathy			1.030	
			(0.043)	
Not Close to Any Party				1.019**
				(0.154)
CDU				0.610
				(0.087)
Greens				1.721**
				(0.291)
Left				1.462
				(0.296)
FDP				1.179
				(0.238)
AfD				1.190
				(0.245)
Other Party				2.860***
				(0.620)
Observations	1,598	1,515	1,598	1,522
Log Likelihood	-1,107.740	-1,043.013	-1,107.227	-1,032.169

Dependent variable: Support for international vaccine solidarity (1 = support, 0 = opposition). Standard errors in parentheses, coefficients show odds ratios. Reference groups: No university education, male, age 18–24, party: Social Democrats (SPD).

*p<0.05;

**p<0.01;

***p<0.001

In line with our hypothesis, we found that empathy predicts higher willingness to share doses internationally (OR = 1.272, SE = .069, *p* < .001). We also found that cosmopolitanism and support for domestic redistribution predict higher support for international dose sharing (cosmopolitanism: OR = 1.279, SE = .061, *p* < .001; support for domestic redistribution: OR = 1.307, SE = .067, *p* < .001). These results held also when we used attitudes toward immigrants (OR = 1.250, SE = .050, *p* < .001, see S9 Table in S1 File) or authoritarianism, a reverse measure (OR = 0.582, SE = .090, *p* = .003, see S11 Table in S1 File), instead of cosmopolitanism.

In our main model, those above age 55 showed less support for vaccine solidarity (OR = 0.581, SE = .363, *p* = .024). However, this finding was not robust to controlling for ideology (OR = 0.788, SE = .481, *p* = .346). Since the reference category (those aged 18–24) was small (4.5% of the sample), we estimated models using a continuous age parameter and found a negative relationship between older age and international vaccine solidarity (*ps*.010, see S12 Table in S1 File). We found the same when we re-estimated the model with a single indicator for whether respondents were aged 55 and above (*ps*.023, see S13 Table in S1 File). Therefore, while we mostly found support for our hypothesis on the role of age, future research should examine whether or not personal risk is a persistent predictor that decreases vaccine solidarity, and how perceptions of personal risk are associated with age.

In only one of our models did we find that conspiratorial thinking predicted higher support for international dose-sharing. However, in most specifications, it was not systematically related to international vaccine solidarity (*ps*.094).

We examined if the role of empathy was conditional on the community conception of respondents—that is, whether the effect differed between non-cosmopolitans and cosmopolitans. The interaction between cosmopolitanism and empathy was not significant (*p* = .491). Empathy’s effect on support for international dose-sharing was positive and significant at all levels of cosmopolitanism. Cosmopolitanism’s effects on international dose-sharing were not significant at the lowest levels of empathy (*p* = .217), but were significant at all other levels of empathy (*ps* < .038). We treat these results with caution given the low number of respondents at the lowest levels of empathy (n = 36).

Finally, we also examined the role of party identification but we did not register hypotheses on these associations. Respondents who identified with the Social Democrats (SPD; winner of the election prior to our data collection) were the reference category. We found, relative to SPD supporters, that respondents who identified with the conservative CDU showed less support for international vaccine solidarity (OR = 0.610, SE = .087, *p* = .003). Individuals who identified with the Green Party, meanwhile, showed more support for dose sharing (OR = 1.721, SE = .291, *p* = .008), reinforcing results found in related work [7].

Our control variables (education, gender, and social class) were not associated with support for international vaccine solidarity.

As per our pre-registration, our analysis focused on the factors associated with support for or opposition to international vaccine solidarity, and therefore we excluded respondents who selected the "*don’t know*" category in the main analysis. We used listwise deletion in case of missing data. To verify the robustness of our results, we also conducted OLS and multinomial logit analyses that tested whether our results held when respondents were not discarded who selected the "don’t know" category of our main outcome. Results were substantively identical, and can be found in S1 File, where we also show the factors associated with a "*don’t know*" response. Additional sample and questionnaire details are also available in S1 File.

## Discussion

In the context of the COVID-19 pandemic, a new normative literature and public debate revolved around the willingness of citizens in HICs to donate vaccines to LICs. Our analysis contributes empirical insights on support for international vaccine solidarity and the characteristics of individuals who exhibit more vaccine solidarity. We see this as an instance where aid decisions might be perceived as zero-sum: any vaccines that go into the arms of people in other countries would not make it into the arms of people in the country.

Based on a population-based survey in Germany, we found that a plurality prefer sharing doses of the COVID-19 vaccine internationally over keeping them in the host country. This is in line with other recent findings [6, 10, 11] and highlights that politicians might have some room to manoeuvre and fulfil international vaccine sharing pledges.

Our result is particularly noteworthy given that international vaccine sharing, at the time of the survey, was not a prominent part of public discourse, which was mostly focused on national vaccine uptake. It is also important to note that almost one in five respondents had no view, leaving room for opinions to crystallise. In sum, there seems to be potential for more international vaccine sharing and for elite communication that increases the salience of the issue, which could mobilize further support [7].

We also showed that those individuals who have been found to generally support foreign aid in the literature are more likely to support vaccine sharing in the current context: for instance, just as cosmopolitans are more supportive of foreign aid, they are more supportive of sharing doses with LICs. Moreover, those who score higher on empathy and left leaning citizens are more inclined to support redistributing vaccines internationally. This suggests that the German public, to the extent they think about vaccine solidarity, treated it like a typical foreign aid issue.

Seemingly in contrast to other work that linked views on domestic redistribution to those on international redistribution [15], we found that citizens who support domestic redistribution also tend to support foreign redistribution. However, this may be a product of using a different level of analysis (while aggregate support for foreign aid was used elsewhere [15], we used individual level data). Moreover, other work [41] also found a positive relationship between support for domestic redistribution and international redistribution at least among some voters (at the individual level). The reasoning is that these voters might support redistribution primarily because they want to see a reduction of inequality rather than because they would gain from redistribution personally. This logic could apply also in the case of international vaccine solidarity.

Although we draw on pre-registered analyses of a large, nationally representative panel survey of a notable case (one of the largest donors of foreign aid), this research includes limitations. A noteworthy limitation is that our cross sectional survey only provides a snapshot of citizens’ attitudes towards international vaccine solidarity at a particular point in time. Citizens’ attitudes on this issue might in fact be very volatile in nature and driven also by context conditions. Our survey was fielded before a major increase of COVID-19 infections in the autumn of 2021 in Germany. It was also fielded before the discovery and spreading of the Omicron variant. Sharing doses internationally is likely to be seen as a different trade-off depending on the infection risks that citizens face and the protection that previous vaccine doses provide. When we fielded the survey, getting a booster shot was less essential for protection (among those who are not in a high-risk group) than in a context in which a new variant is spreading. We believe that our results still include an important message for public policy. COVID-19 infections are apt to rise and fall repeatedly over the long run, new variants are likely to appear, and in fact, we might face pandemics resulting from different viruses entirely. Our findings show that there is substantial public support among citizens to share doses internationally at least when infection rates are at a modest level and falling. Moreover, while levels of support for dose sharing might change as a result of the domestic risk situation, we argue that the factors that shape citizens’ views on this issue are likely to remain the same—though the magnitude of their effects is of course likely to vary. This is important for the public debate, as it tells us how citizens understand the topic, where support for this policy comes from, and where opposition is likely to be large.

The role of context conditions has not been the focus of our analysis, but it is undoubtedly an important one. Future research should analyse the volatility of public support for an international redistribution of medical supplies such as vaccine doses and what role domestic infections play for the willingness of citizens to share their medical supply internationally.

Another limitation relates to the measures that we employed in our analysis. We relied on single-item measures for several complex concepts (e.g., support for vaccine solidarity, empathy, cosmopolitanism) rather than multi-item scales. Our robustness checks reinforced our results, but future research could use multi-item batteries in order for the analysis to better capture individual differences. Future research could also collect data on support for foreign aid side by side with support for international vaccine solidarity. Moreover, while we used the literature on public support for foreign aid to derive hypotheses, our study does not test the correlation between citizens’ views on both issues.

Finally, it should be noted that the extent to which COVID-19 posed a real or perceived risk and affected citizens is likely to vary in ways beyond the issues we could account for. Individuals who consider themselves to be less at risk or who are less affected—for other reasons—might be more willing to share vaccine shots. Future research might address these factors in a more detailed fashion.

## Supporting information

S1 File Contains all supporting tables. (PDF)
