# Supplementary material for: Correlates of support for international vaccine solidarity during the COVID-19 pandemic: Cross-sectional survey evidence from Germany
Source: PLoS One. 2023 Jun 23;18(6):e0287257. doi: 10.1371/journal.pone.0287257 (PMC10289341; doi:10.1371/journal.pone.0287257)
Supplement: S1 File — (PDF) [file pone.0287257.s001.pdf]

Supplementary Information

Table S1: Demographics

Variable	n	Sample%
Gender	*2,019*	
Male	1,004	49.70
Female	1,015	50.30
Age	*2,019*	
18-24	91	4.50
25-44	599	29.70
45-54	312	15.50
55+	1,017	50.40
Education	*2,019*	
No university	1,405	69.60
University	547	27.10
NA	67	3.30

Questionnaire

International Vaccine Solidarity

Coping with the Covid 19 pandemic requires difficult decisions. By the end of September, about 64% of people eligible for vaccination had been vaccinated at least once. What do you think is the more important priority now for the use of Germany's vaccine stocks: offering a third vaccine dose ("booster vaccination") to people in Germany or giving vaccine stocks for first and second vaccine doses to less developed countries?

<1> Third vaccination dose ("booster vaccination") for people in Germany.

<2> Giving vaccine stocks for first and second vaccine doses to less developed countries.

<99> Don't know / don't want to answer

Cosmopolitanism

Globalization threatens Germany's identity.

<5> Strongly agree

<4> Agree

<3> Neither agree nor disagree

<2> Disagree

<1> Strongly disagree

Empathy

Please indicate how well the following statement describes you: When I see a person being taken advantage of, I want to protect them.

<1> (1) Does NOT describe me well.

<2> (2)

<3> (3)

<4> (4)

<5> (5) Describes me well

Conspiratorial Thinking

Much of our lives are being controlled by plots hatched in secret places.

<5> Strongly agree

<4> Agree

<3> Neither agree nor disagree

<2> Disagree

<1> Strongly disagree

Even though we live in a democracy, a few people will always run things anyway.

Same scale as above

The people who really ’run’ the country are not known to the voter.

Same scale as above

Support for Domestic Redistribution

Government should do more to reduce income inequality.

<5> Strongly agree

<4> Agree

<3> Neither agree nor disagree

<2> Disagree

<1> Strongly disagree

Authoritarianism

Would you say that it is more important for a child to be INDEPENDENT or RESPECTFUL OF THEIR ELDERS?

<1> Independent

<2> Respectful of their elders

Would you say that it is more important for a child to be OBEDIENT or SELF-RELIANT?

<1> Obedient

<2> Self-reliant

Would you say that it is more important for a child to be ADAPTABLE or DISCIPLINED?

<1> Adaptable

<2> Disciplined

Would you say that it is more important for a child to be CURIOUS or GOOD MANNERED?

<1> Curious

<2> Good mannered

Pro-Immigrant Affect

To what extent do you have negative or positive feelings when you think about immigrants?

<1> -3 Very negative feelings

<2> -2

<3> -1

<4> 0 Neutral feelings

<5> 1

<6> 2

<7> 3 Very positive feelings

Social Class

Imagine that the scale below represents the society in which you live. At the upper end of the scale are the people who are doing best in our society: they have the most resources and the best jobs. At the lower end of the scale are the people in our society who are doing the worst: they have the fewest resources, no jobs, or jobs that no one wants or respects. Now think about yourself or your family (if you are close to them). Please indicate where you think you or your family would be on this scale.

<9> (9) I am among the people who are doing best

<8> (8)

<7> (7)

<6> (6)

<5> (5)

<4> (4)

<3> (3)

<2> (2)

<1> (1) I am one of the people who are the worst off

Left-Right Self-Placement

In politics, people talk about left and right. Where do you stand? Please indicate your personal position on a scale from 0 (very left) to 10 (very right).

<0> 0 - Very left

<1> 1

<2> 2

<3> 3

<4> 4

<5> 5

<6> 6

<7> 7

<8> 8

<9> 9

<10> 10 - Very right

<98> Do not know

Party Identification

Which party do you feel closest to?

<0> I am not close to any party

<1> CDU/CSU

<2> SPD

<3> The Greens

<4> The Left

<5> FDP

<6> AfD

<7> Other party

<98> Do not know

Professional Educational Attainment (provided by YouGov)

<1> no degree: coded as no university education (0)

<2> still in vocational training: coded as no university education (0)

<3> still studying: coded as university education (1)

<4> vocational degree of equivalent training: coded as no university education (0)

<5> university degree (including degrees from a university of applied sciences, i.e. Fachhochschulabschluss): coded as university education (1)

<777> no answer: excluded

Table S2: Descriptive Statistics for Key Constructs

	M	SD	Min	Max
Cosmopolitanism	3.10	1.20	1.00	5.00
Authoritarianism	1.45	0.30	1.00	2.00
Immigrant	3.64	1.46	1.00	7.00
Empathy	3.92	0.95	1.00	5.00
Conspiratorial Thinking	3.01	1.11	1.00	5.00
Economic Conservatism	3.93	1.04	1.00	5.00
Left-Right Self-Placement	4.83	1.78	0.00	10.00
Social Class	5.25	1.62	1.00	9.00

Table S3: Main results of OLS regressions that predict international vaccine solidarity (1=support for international dose sharing, 0=opposition to international dose sharing)

	Dependent variable:			
	International Vaccine Solidarity			
	(1)	(2)	(3)	(4)
Empathy	0.055***	0.057***	0.047*	0.054***
	(0.013)	(0.013)	(0.023)	(0.013)
Cosmopolitanism	0.055***	0.051***	0.043	0.046***
	(0.011)	(0.011)	(0.030)	(0.011)
Support for Domestic Redistribution	0.061***		0.061***	0.050***
	(0.012)		(0.012)	(0.013)
Left-Right Self-Placement		−0.035***		
		(0.007)		
Age 25-44	−0.041	0.021	−0.041	−0.053
	(0.053)	(0.057)	(0.053)	(0.053)
Age 45-54	−0.044	0.034	−0.044	−0.031
	(0.059)	(0.063)	(0.059)	(0.059)
Age 55+	−0.121*	−0.056	−0.121*	−0.097
	(0.053)	(0.057)	(0.053)	(0.052)
Conspiratorial Thinking	0.021	0.034**	0.021	0.017
	(0.013)	(0.013)	(0.013)	(0.013)
Cosmopolitanism * Empathy			0.004	
			(0.010)	
Female	0.016	0.005	0.016	0.012
	(0.024)	(0.025)	(0.024)	(0.025)
University	0.039	0.012	0.039	0.036
	(0.028)	(0.029)	(0.028)	(0.029)
Social Class	0.003	−0.001	0.003	0.008
	(0.008)	(0.008)	(0.008)	(0.008)
CDU/CSU				−0.119**
				(0.028)
Not Close to Any Party				0.005
				(0.040)
Greens				0.114**
				(0.044)
Left				0.083
				(0.056)
FDP				0.039
				(0.058)
AfD				0.034
				(0.060)
Other Party				0.212***
				(0.056)
Constant	0.067	0.417***	0.093	0.124
	(0.104)	(0.102)	(0.119)	(0.109)
Observations	1,598	1,515	1,598	1,522
R^2	0.057	0.053	0.057	0.089

Reference categories: No university education, Age 18-24, male, party: Social Democrats (SPD)

Note: Standard errors in parentheses. $^{*}p<0.05$; $^{**}p<0.01$; $^{***}p<0.001$

Table S4: Replication of model 1 of main analyses using multinomial logistic regression. Reference category = keep doses for Germans.

	Dependent variable:	
	Share Doses	DK
Cosmopolitanism	0.251^{***}	0.073
	(0.050)	(0.064)
Empathy	0.246^{***}	0.077
	(0.058)	(0.070)
Age 25-44	−0.266	−0.228
	(0.244)	(0.299)
Age 45-54	−0.284	−0.332
	(0.269)	(0.331)
Age 55+	-0.615^{*}	-0.694^{*}
	(0.239)	(0.295)
Conspiratorial Thinking	0.089	0.528^{***}
	(0.055)	(0.072)
Support for Domestic Redistribution	0.278^{***}	−0.040
	(0.054)	(0.067)
Female	0.067	0.376^{**}
	(0.107)	(0.136)
University	0.146	−0.263
	(0.124)	(0.168)
Social Class	0.017	−0.043
	(0.035)	(0.043)
Constant	-1.900^{***}	-1.895^{***}
	(0.460)	(0.568)
Observations	1,950	1,950

Reference categories: No university education, Age 18-24, male, party: Social Democrats (SPD)
Note: *p<0.05; **p<0.01; ***p<0.001

Table S5: Replication of model 2 of main analyses using multinomial logistic regression. Reference category = keep doses for Germans.

	Dependent variable:	
	Share Doses	DK
Cosmopolitanism	0.226***	0.068
	(0.051)	(0.068)
Empathy	0.254***	0.042
	(0.060)	(0.076)
Age 25-44	0.041	−0.225
	(0.256)	(0.322)
Age 45-54	0.128	−0.132
	(0.285)	(0.358)
Age 55+	−0.275	−0.622*
	(0.251)	(0.317)
Conspiratorial Thinking	0.143*	0.524***
	(0.057)	(0.077)
Left-Right Self-Placement	−0.145***	−0.034
	(0.033)	(0.042)
Female	0.008	0.365*
	(0.110)	(0.146)
University	0.042	−0.305
	(0.127)	(0.178)
Social Class	0.002	−0.029
	(0.036)	(0.046)
Constant	−0.414	−1.955***
	(0.451)	(0.582)
Observations	1,823	1,823

Reference categories: No university education, Age 18-24, male, party: Social Democrats (SPD)

Note: *p<0.05; **p<0.01; ***p<0.001

Table S6: Replication of model 3 of main analyses using multinomial logistic regression. Reference category = keep doses for Germans.

	Dependent variable:	
	Share Doses	DK
Cosmopolitanism	0.164	−0.033
	(0.137)	(0.167)
Empathy	0.188	0.011
	(0.102)	(0.116)
Age 25-44	−0.266	−0.225
	(0.244)	(0.299)
Age 45-54	−0.287	−0.334
	(0.269)	(0.332)
Age 55+	−0.617**	−0.694*
	(0.239)	(0.295)
Conspiratorial Thinking	0.092	0.531***
	(0.055)	(0.073)
Support for domestic redistribution	0.275***	−0.043
	(0.054)	(0.067)
Female	0.070	0.380**
	(0.107)	(0.136)
University	0.145	−0.265
	(0.125)	(0.168)
Social Class	0.017	−0.043
	(0.035)	(0.043)
Cosmopolitanism * Empathy	0.031	0.038
	(0.045)	(0.055)
Constant	−1.731***	−1.712**
	(0.525)	(0.625)
Observations	1,950	1,950

Reference categories: No university education, Age 18-24, male, party: Social Democrats (SPD)
Note: *p<0.05; **p<0.01; ***p<0.001

Table S7: Replication of model 4 of main analyses using multinomial logistic regression. Reference category = keep doses for Germans.

	Dependent variable:	
	Share Doses	DK
Cosmopolitanism	0.214^{***}	0.101
	(0.053)	(0.069)
Empathy	0.246^{***}	0.091
	(0.060)	(0.075)
Age 25-44	−0.199	−0.060
	(0.248)	(0.320)
Age 45-54	−0.119	−0.074
	(0.276)	(0.355)
Age 55+	−0.429	−0.354
	(0.244)	(0.315)
Conspiratorial Thinking	0.068	0.407^{***}
	(0.058)	(0.078)
Support for Domestic Redistribution	0.242^{***}	0.007
	(0.058)	(0.074)
Female	0.046	0.462^{**}
	(0.112)	(0.147)
University	0.139	−0.082
	(0.133)	(0.180)
Social Class	0.041	0.023
	(0.037)	(0.048)
CDU/CSU	-0.503^{**}	-0.551^{*}
	(0.167)	(0.248)
Not Close to Any Party	0.02	0.407
	(0.179)	(0.237)
Greens	0.536^{*}	0.083
	(0.206)	(0.316)
Left	0.353	0.546
	(0.257)	(0.341)
FDP	0.147	0.664^{*}
	(0.256)	(0.317)
AfD	0.160	1.268^{***}
	(0.260)	(0.285)
Other Party	1.041^{***}	1.014^{**}
	(0.279)	(0.344)
Constant	-1.838^{***}	-2.609^{***}
	(0.500)	(0.643)
Observations	1,740	1,740

Reference categories: No university education, Age 18-24, male, party: Social Democrats (SPD)
Note: *p<0.05; **p<0.01; ***p<0.001

Table S8: Replication of main analyses using pro-immigrant affect instead of cosmopolitanism.

	Dependent variable:			
	International Vaccine Solidarity			
	(1)	(2)	(3)	(4)
Pro-Immigrant Affect	0.049***	0.041***	0.077**	0.042***
	(0.009)	(0.010)	(0.025)	(0.010)
Empathy	0.045***	0.048***	0.068**	0.046***
	(0.013)	(0.014)	(0.023)	(0.013)
Age 25-44	−0.039	0.018	−0.038	−0.050
	(0.053)	(0.057)	(0.053)	(0.053)
Age 45-54	−0.041	0.031	−0.039	−0.030
	(0.059)	(0.063)	(0.059)	(0.059)
Age 55+	−0.123*	−0.062	−0.123*	−0.100
	(0.052)	(0.057)	(0.052)	(0.052)
Conspiratorial Thinking	0.012	0.022	0.012	0.008
	(0.012)	(0.012)	(0.012)	(0.012)
Support for Domestic Redistribution	0.053***		0.053***	0.043***
	(0.012)		(0.012)	(0.013)
Left-Right Self-Placement		−0.029***		
		(0.008)		
Female	0.010	0.001	0.012	0.006
	(0.024)	(0.025)	(0.024)	(0.025)
University	0.024	0.004	0.024	0.027
	(0.028)	(0.029)	(0.028)	(0.029)
Social Class	0.0001	−0.003	0.0002	0.005
	(0.008)	(0.008)	(0.008)	(0.008)
Pro-Immigrant Affect * Empathy			−0.009	
			(0.008)	
CDU/CSU				−0.113**
				(0.038)
Not Close to Any Party				0.024
				(0.040)
Greens				0.109*
				(0.044)
Left				0.072
				(0.056)
FDP				0.048
				(0.058)
AfD				0.070
				(0.061)
Other Party				0.205***
				(0.056)
Constant	0.148	0.456***	0.079	0.175
	(0.098)	(0.100)	(0.113)	(0.104)
Observations	1,598	1,515	1,598	1,522
R^2	0.060	0.052	0.061	0.090

Reference categories: No university education, Age 18-24, male, party: Social Democrats (SPD)
Note: *p<0.05; **p<0.01; ***p<0.001

Table S9: Replication of main analyses using pro-immigrant affect instead of cosmopolitanism, logistic regression (coefficients are odds ratios).

	Dependent variable:			
	International Vaccine Solidarity			
	(1)	(2)	(3)	(4)
Pro-Immigrant Affect	1.250***	1.197***	1.383**	1.217***
	(0.050)	(0.050)	(0.143)	(0.010)
Empathy	1.220***	1.231***	1.329**	1.234***
	(0.066)	(0.069)	(0.129)	(0.013)
Age 25-44	0.845	1.088	0.845	0.797
	(0.164)	(0.220)	(0.164)	(0.053)
Age 45-54	0.833	1.157	0.838	0.873
	(0.175)	(0.253)	(0.176)	(0.059)
Age 55+	0.580*	0.765	0.581*	0.634
	(0.111)	(0.152)	(0.111)	(0.052)
Conspiratorial Thinking	1.059	1.106	1.057	1.037
	(0.054)	(0.057)	(0.053)	(0.012)
Support for Domestic Redistribution	1.260***		1.262***	1.218***
	(0.064)		(0.065)	(0.013)
Left-Right Self-Placement		0.879***		
		(0.030)		
Female	1.050	1.007	1.054	1.030
	(0.102)	(0.100)	(0.102)	(0.025)
University	1.126	1.029	1.127	1.152
	(0.127)	(0.117)	(0.127)	(0.029)
Social Class	0.998	0.983	0.999	1.024
	(0.035)	(0.035)	(0.035)	(0.008)
Pro-Immigrant Affect * Empathy			0.966	
			(0.034)	
CDU/CSU				0.628**
				(0.038)
Not Close to Any Party				1.113
				(0.040)
Greens				1.685*
				(0.044)
Left				1.386
				(0.056)
FDP				1.233
				(0.058)
AfD				1.396
				(0.061)
Other Party				2.785***
				(0.056)
Observations	1,598	1,515	1,598	1,522
Log Likelihood	−1,104.710	−1,043.824	−1,104.191	−1,030.67

Reference categories: No university education, Age 18-24, male, party: Social Democrats (SPD)
Note: *p<0.05; **p<0.01; ***p<0.001

Table S10: Main results of logistic regressions that predict international vaccine solidarity (1=support for international dose sharing, 0=opposition to international dose sharing)

	Dependent variable:			
	International Vaccine Solidarity			
	(1)	(2)	(3)	(4)
Authoritarianism	-0.124^{**}	-0.096^{*}	-0.274^{*}	-0.073
	(0.042)	(0.044)	(0.128)	(0.043)
Empathy	0.052^{***}	0.053^{***}	-0.025	0.052^{***}
	(0.013)	(0.014)	(0.063)	(0.013)
Age 25-44	-0.060	0.009	-0.063	-0.064
	(0.054)	(0.058)	(0.054)	(0.054)
Age 45-54	-0.071	0.013	-0.070	-0.051
	(0.060)	(0.064)	(0.060)	(0.060)
Age 55+	-0.147^{**}	-0.075	-0.149^{**}	-0.116^{*}
	(0.053)	(0.057)	(0.053)	(0.053)
Conspiratorial Thinking	0.002	0.016	0.001	0.001
	(0.012)	(0.012)	(0.012)	(0.012)
Support for Domestic Redistribution	0.056^{***}		0.056^{***}	0.045^{***}
	(0.012)		(0.012)	(0.013)
Left-Right Self-Placement		-0.035^{***}		
		(0.008)		
Female	0.010	0.001	0.010	0.008
	(0.024)	(0.025)	(0.024)	(0.025)
University	0.036	0.012	0.036	0.036
	(0.029)	(0.029)	(0.029)	(0.029)
Social Class	0.004	-0.0003	0.003	0.008
	(0.008)	(0.008)	(0.008)	(0.008)
Authoriarianism * Empathy			0.052	
			(0.042)	
CDU/CSU				-0.120^{**}
				(0.038)
Not Close to Any Party				0.012
				(0.041)
Greens				0.123^{**}
				(0.044)
Left				0.082
				(0.056)
FDP				0.032
				(0.059)
AfD				0.013
				(0.060)
Other Party				0.206^{***}
				(0.056)
Constant	0.466^{***}	0.736^{***}	0.691^{**}	0.406^{***}
	(0.116)	(0.110)	(0.216)	(0.120)
Observations	1,598	1,515	1,598	1,522
R^2	0.047	0.044	0.048	0.080

Reference categories: No university education, Age 18-24, male, party: Social Democrats (SPD)

Note: *p<0.05; **p<0.01; ***p<0.001

Table S11: Replication of main analyses using authoritarianism instead of cosmopolitanism, logistic regressions (coefficients are odds ratios).

	Dependent variable:			
	International Vaccine Solidarity			
	(1)	(2)	(3)	(4)
Authoritarianism	0.582**	0.658*	0.319*	0.720
	(0.090)	(0.106)	(0.109)	(0.117)
Empathy	1.249***	1.252***	0.915	1.258***
	(0.067)	(0.070)	(0.199)	(0.070)
Age 25-44	0.764	1.042	0.755	0.743
	(0.148)	(0.211)	(0.146)	(0.147)
Age 45-54	0.727	1.070	0.730	0.789
	(0.152)	(0.234)	(0.153)	(0.169)
Age 55+	0.524**	0.723	0.518**	0.588*
	(0.100)	(0.144)	(0.099)	(0.114)
Conspiratorial Thinking	1.007	1.072	1.006	1.001
	(0.049)	(0.054)	(0.049)	(0.053)
Support for Domestic Redistribution	1.271***		1.223***	0.045***
	(0.065)		(0.065)	(0.066)
Left-Right Self-Placement		0.856***		
		(0.028)		
Female	1.048	1.005	1.046	1.036
	(0.101)	(0.100)	(0.100)	(0.104)
University	1.176	1.058	1.177	1.192
	(0.132)	(0.119)	(0.132)	(0.141)
Social Class	1.016	0.998	1.015	1.035
	(0.035)	(0.035)	(0.035)	(0.038)
Authoriarianism * Empathy			1.233	
			(0.193)	
CDU/CSU				0.611**
				(0.087)
Not Close to Any Party				1.054
				(0.158)
Greens				1.794**
				(0.303)
Left				1.439
				(0.290)
FDP				1.145
				(0.231)
AfD				1.069
				(0.218)
Other Party				2.761***
				(0.598)
Observations	1,598	1,515	1,598	1,522
Log Likelihood	−1,116.856	−1,051.042	−1,116.592	−1,040.204

Reference categories: No university education, Age 18-24, male, party: Social Democrats (SPD)
Note: *p<0.05; **p<0.01; ***p<0.001

Table S12: Replication of main analyses using a continuous age variable, logistic regression (coefficients are odds ratios).

	Dependent variable:			
	International Vaccine Solidarity			
	(1)	(2)	(3)	(4)
Cosmopolitanism	1.272***	1.247***	1.162	1.232***
	(0.061)	(0.061)	(0.136)	(0.062)
Empathy	1.281***	1.296***	1.206	1.281***
	(0.069)	(0.073)	(0.109)	(0.072)
Age	0.987***	0.990**	0.987***	0.991**
	(0.003)	(0.003)	(0.003)	(0.003)
Conspiratorial Thinking	1.090	1.155*	1.093	1.074
	(0.086)	(0.063)	(0.058)	(0.060)
Support for Domestic Redistribution	1.314***		1.309***	1.257***
	(0.067)		(0.067)	(0.069)
Left-Right Self-Placement		0.861***		
		(0.028)		
Female	1.087	1.038	1.088	1.069
	(0.105)	(0.103)	(0.105)	(0.108)
University	1.153	1.045	1.152	1.159
	(0.129)	(0.118)	(0.129)	(0.137)
Social Class	1.016	0.990	1.015	1.034
	(0.034)	(0.035)	(0.034)	(0.037)
Cosmopolitanism * Empathy			1.032	
			(0.086)	
CDU				0.607**
				(0.154)
Not Close To Any Party				1.023
				(0.179)
Greens				1.713**
				(0.290)
Left				1.424
				(0.288)
FDP				1.173
				(0.237)
AfD				1.171
				(0.240)
Other Party				2.720***
				(0.589)
Observations	1,598	1,515	1,598	1,522
Log Likelihood	−1,105.709	−1,042.224	−1,105.150	−1,032.432

Reference categories: No university education, Age 18-24, male, party: Social Democrats (SPD)

Note: *p<0.05; **p<0.01; ***p<0.001

Table S13: Replication of main analyses using a single indicator for whether a respondent is aged 55 or older, logistic regression (coefficients are odds ratios).

	Dependent variable:			
	International Vaccine Solidarity			
	(1)	(2)	(3)	(4)
Cosmopolitanism	1.280***	1.251***	1.178	1.237***
	(0.061)	(0.061)	(0.137)	(0.062)
Empathy	1.268***	1.286***	1.199	1.274***
	(0.068)	(0.072)	(0.108)	(0.072)
Age 55+	0.692***	0.709**	0.691***	0.769*
	(0.068)	(0.072)	(0.068)	(0.079)
Conspiratorial Thinking	1.097	1.160**	1.100	1.077
	(0.058)	(0.063)	(0.058)	(0.060)
Support for Domestic Redistribution	1.309***		1.254***	0.227***
	(0.067)		(0.066)	(0.069)
Left-Right Self-Placement		0.858***		
		(0.028)		
Female	1.072	1.026	1.074	1.060
	(0.103)	(0.102)	(0.104)	(0.107)
University	1.193	1.058	1.193	1.181
	(0.132)	(0.119)	(0.133)	(0.139)
Social Class	1.017	0.991	1.016	1.035
	(0.034)	(0.035)	(0.034)	(0.037)
Cosmopolitanism * Empathy			1.030	
			(0.042)	
CDU				0.603**
				(0.085)
Not Close To Any Party				1.020
				(0.154)
Greens				1.719**
				(0.291)
Left				1.445
				(0.292)
FDP				1.187
				(0.240)
AfD				1.175
				(0.241)
Other Party				2.782***
				(0.601)
Observations	1,598	1,515	1,598	1,522
Log Likelihood	−1,108.594	−1,043.057	−1,108.086	−1,033.484

Reference categories: No university education, Age 18-24, male, party: Social Democrats (SPD)

Note: *p<0.05; **p<0.01; ***p<0.001

Table S14: Replication of main analyses removing respondents who took less than 5 minutes to do the survey.

	Dependent variable:			
	International Vaccine Solidarity			
	(1)	(2)	(3)	(4)
Cosmopolitanism	1.264***	1.235***	1.241	1.228***
	(0.063)	(0.063)	(0.153)	(0.064)
Empathy	1.261***	1.257***	1.245*	1.263***
	(0.072)	(0.075)	(0.117)	(0.075)
Age 25-44	0.543	0.627	0.543	0.488*
	(0.133)	(0.160)	(0.134)	(0.122)
Age 45-54	0.525	0.620	0.526	0.559
	(0.135)	(0.165)	(0.135)	(0.146)
Age 55+	0.366**	0.417*	0.366**	0.404**
	(0.089)	(0.105)	(0.089)	(0.100)
Conspiratorial Thinking	1.134*	1.184**	1.134*	1.114
	(0.062)	(0.067)	(0.062)	(0.064)
Support for Domestic Redistribution	1.253***		1.252***	1.210**
	(0.068)		(0.068)	(0.071)
Left-Right Self-Placement		0.851***		
		(0.031)		
Female	1.091	1.014	1.091	1.098
	(0.110)	(0.106)	(0.110)	(0.116)
University	1.340*	1.175	1.339*	1.244
	(0.162)	(0.144)	(0.162)	(0.159)
Social Class	1.002	0.993	1.002	1.028
	(0.036)	(0.037)	(0.036)	(0.040)
Cosmopolitanism * Empathy			1.006	
			(0.044)	
CDU				0.774
				(0.117)
Not Close to Any Party				1.105
				(0.173)
Greens				2.117***
				(0.373)
Left				1.860*
				(0.398)
FDP				1.297
				(0.273)
AfD				1.289
				(0.275)
Other Party				3.305***
				(0.746)
Observations	1,509	1,434	1,509	1,438
Log Likelihood	−1,015.567	−951.950	−1,015.493	−947.275

Reference categories: No university education, Age 18-24, male, party: Social Democrats (SPD)

Note: *p<0.05; **p<0.01; ***p<0.001
